# 3-Hy­droxy-1-[(morpholin-4-yl)meth­yl]pyridazin-6(1*H*)-one

**DOI:** 10.1107/S1600536813010477

**Published:** 2013-04-20

**Authors:** P. R. Santhi, G. Selvanathan, G. Poongothai, T. Srinivasan, D. Velmurugan

**Affiliations:** a Department of Chemistry, AVC College (Autonomous), Mannampandal 609 305, Tamilnadu, India; b Department of Chemistry, Government Arts College for Men (Autonomous), Nandanam, Chennai-35, Tamilnadu, India; c Centre of Advanced Study in Crystallography and Biophysics, University of Madras, Guindy Campus, Chennai 600 025, India

## Abstract

In the title compound, C_9_H_13_N_3_O_3_, the morpholine ring adopts a chair conformation and its mean plane makes a dihedral angle of 68.00 (11)° with the pyridazine ring. The carbonyl O atom deviates from the plane of the pyridazine ring by 0.0482 (12) Å. An intra­molecular C—H⋯O hydrogen bond occurs. In the crystal, mol­ecules are linked by O—H⋯O and C—H⋯O hydrogen bonds, forming chains along [1-10].

## Related literature

For the biological activity of morpholine derivatives, see: Lan *et al.* (2010 ▶); Raparti *et al.* (2009 ▶). For a related structure, see: Wang *et al.* (2012 ▶).
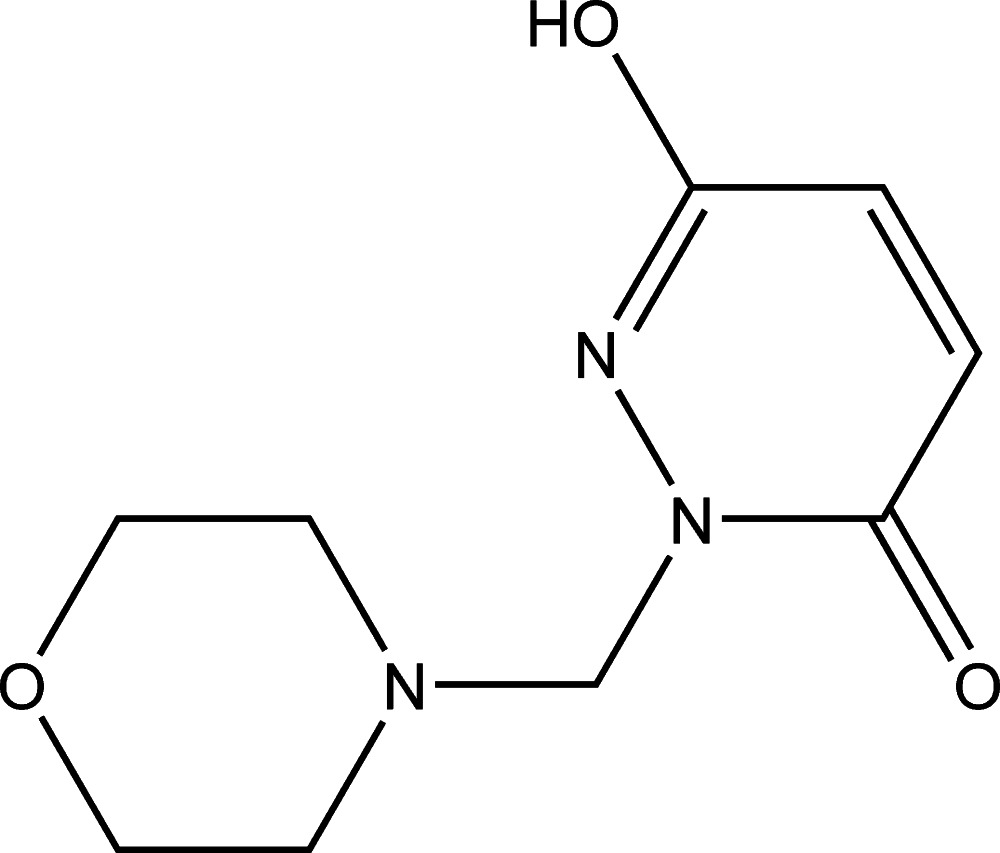


## Experimental

### 

#### Crystal data

C_9_H_13_N_3_O_3_
*M* *_r_* = 211.22
Triclinic, 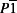

*a* = 5.2110 (3) Å
*b* = 5.4165 (4) Å
*c* = 18.4544 (12) Å
α = 87.232 (2)°
β = 83.993 (6)°
γ = 80.862 (4)°
*V* = 511.18 (6) Å^3^
*Z* = 2
Mo *K*α radiation
μ = 0.11 mm^−1^
*T* = 293 K
0.30 × 0.25 × 0.20 mm

#### Data collection

Bruker SMART APEXII area-detector diffractometer
Absorption correction: multi-scan (*SADABS*; Bruker, 2008 ▶) *T*_min_ = 0.969, *T*_max_ = 0.979
8839 measured reflections
2530 independent reflections
1679 reflections with *I* > 2σ(*I*)
*R*_int_ = 0.027

#### Refinement

*R*[*F* ^2^ > 2σ(*F* ^2^)] = 0.051
*wR*(*F* ^2^) = 0.158
*S* = 1.07
2530 reflections
136 parameters
H-atom parameters constrained
Δρ_max_ = 0.31 e Å^−3^
Δρ_min_ = −0.23 e Å^−3^

### 

Data collection: *APEX2* (Bruker, 2008 ▶); cell refinement: *SAINT* (Bruker, 2008 ▶); data reduction: *SAINT*; program(s) used to solve structure: *SHELXS97* (Sheldrick, 2008 ▶); program(s) used to refine structure: *SHELXL97* (Sheldrick, 2008 ▶); molecular graphics: *ORTEP-3 for Windows* (Farrugia, 2012 ▶); software used to prepare material for publication: *SHELXL97* and *PLATON* (Spek, 2009 ▶).

## Supplementary Material

Crystal structure: contains datablock(s) global, I. DOI: 10.1107/S1600536813010477/pv2627sup1.cif

Structure factors: contains datablock(s) I. DOI: 10.1107/S1600536813010477/pv2627Isup2.hkl

Supplementary material file. DOI: 10.1107/S1600536813010477/pv2627Isup3.cml

Additional supplementary materials:  crystallographic information; 3D view; checkCIF report

## Figures and Tables

**Table 1:** Hydrogen-bond geometry (Å, °)

*D*—H⋯*A*	*D*—H	H⋯*A*	*D*⋯*A*	*D*—H⋯*A*
O1—H1⋯O2^i^	0.82	1.77	2.5777 (16)	167
C2—H2⋯O1^ii^	0.93	2.55	3.478 (2)	175
C5—H5*A*⋯O2	0.97	2.44	2.772 (2)	100

Symmetry codes: (i) 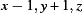; (ii) 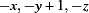.

**Fig. 1.**
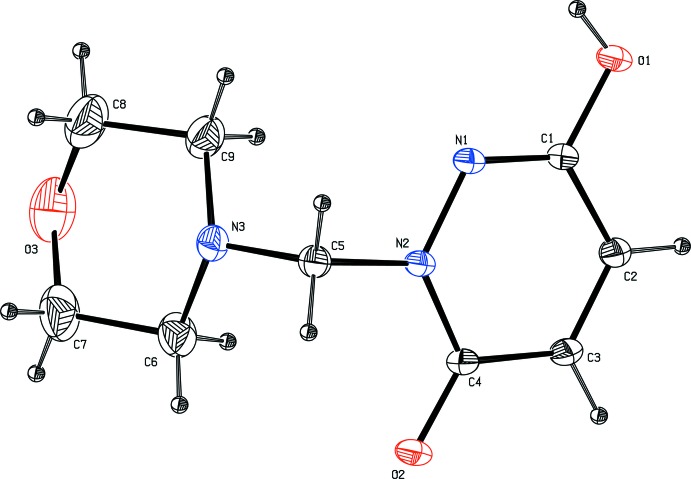
The molecular structure of the title compound, showing displacement ellipsoids drawn at the 30% probability level. H atoms are presented as small spheres of arbitrary radius.

**Fig. 2.**
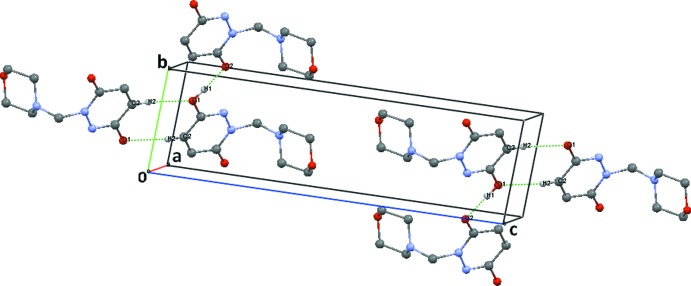
The crystal packing of the title compound viewed down a axis. H-atoms not involved in H-bonds have been excluded for clarity.

## supplementary crystallographic information

### Comment

Morpholine derivatives possess anticancer and antimicrobial activities (Lan *et al.*, 2010; Raparti *et al.*, 2009). In the title compound (Fig. 1), the morpholine ring (N3/O3/C6-C9) adopts a *chair* conformation. The morpholine ring makes a dihedral angle of 68.00 (11)° with the pyridazin ring (N1/N2/C1-C4). The hydroxyl oxygen atom O1 attached with the pyridazin ring deviates by 0.0242 (13)Å. The oxygen atom O2 attached with the pyridazin ring deviates by 0.0482 (12)Å. The packing of the crystal is stabilised by intermolcular O—H···O hydrogen bonds and weak intramolecular C—H···O hydrogen bonds (Fig. 2 & Table 1).

### Experimental

The new Mannich base morpholino methyl maleic hydrazide(MMMH) was synthesised by introducing morpholino methyl moiety in place of active hydrogen atom attached to nitrogen of maleic hydrazide through Mannich reaction. An equimolar mixture of maleic hydrazide (11.20 g), formaldehyde (3.00 g) and morpholine (8.7 g) was dissolved in 400 ml of ethanol and refluxed for about 5 hours. The formation of the product MMMH and the completion of the reaction was identified by the formation of a clear solution. The resulting solution was concentrated to 200 ml by distillation under reduced pressure. The concentrate on cooling yielded a colourless crystalline solid, the crude product (20.6g) that was first washed with ethanol and then ether and dried in vacuum oven. The compound MMMH was dissolved in hot ethanol and the homogeneous solution was allowed to evaporate slowly. After two weeks the colourless crystalline solid separated out which was washed with minimum amount of ethanol and then dried in a vaccum oven; a crystal was chosen for X-ray diffraction studies from this sample.

### Refinement

All C-bound H atoms were positioned geometrically and refined using a riding model, with C—H = 0.93 and 0.97 Å, for aryl and methylene H-atoms, respectively. The hydroxyl H-atoms were included at geometrically calculated positions with O—H = 0.82 Å. The H-atoms are constrained to ride on their parent atoms, with *U*_iso_(H) = 1.2 times *U*_eq_(C/O).

### Crystal data

**Table:**

C_9_H_13_N_3_O_3_	*Z* = 2
*M**_r_* = 211.22	*F*(000) = 224
Triclinic, *P*1	*D*_x_ = 1.372 Mg m^−^^3^
Hall symbol: -P 1	Mo *K*α radiation, λ = 0.71073 Å
*a* = 5.2110 (3) Å	Cell parameters from 2530 reflections
*b* = 5.4165 (4) Å	θ = 1.1–28.4°
*c* = 18.4544 (12) Å	µ = 0.11 mm^−^^1^
α = 87.232 (2)°	*T* = 293 K
β = 83.993 (6)°	Block, colourless
γ = 80.862 (4)°	0.30 × 0.25 × 0.20 mm
*V* = 511.18 (6) Å^3^	

### Data collection

**Table:**

Bruker SMART APEXII area-detector diffractometer	2530 independent reflections
Radiation source: fine-focus sealed tube	1679 reflections with *I* > 2σ(*I*)
Graphite monochromator	*R*_int_ = 0.027
ω and φ scans	θ_max_ = 28.4°, θ_min_ = 1.1°
Absorption correction: multi-scan (*SADABS*; Bruker, 2008)	*h* = −6→6
*T*_min_ = 0.969, *T*_max_ = 0.979	*k* = −7→7
8839 measured reflections	*l* = −24→24

### Refinement

**Table:**

Refinement on *F*^2^	Primary atom site location: structure-invariant direct methods
Least-squares matrix: full	Secondary atom site location: difference Fourier map
*R*[*F*^2^ > 2σ(*F*^2^)] = 0.051	Hydrogen site location: inferred from neighbouring sites
*wR*(*F*^2^) = 0.158	H-atom parameters constrained
*S* = 1.07	*w* = 1/[σ^2^(*F*_o_^2^) + (0.0734*P*)^2^ + 0.1237*P*] where *P* = (*F*_o_^2^ + 2*F*_c_^2^)/3
2530 reflections	(Δ/σ)_max_ < 0.001
136 parameters	Δρ_max_ = 0.31 e Å^−^^3^
0 restraints	Δρ_min_ = −0.23 e Å^−^^3^

### Special details

**Table:**

Geometry. All esds (except the esd in the dihedral angle between two l.s. planes) are estimated using the full covariance matrix. The cell esds are taken into account individually in the estimation of esds in distances, angles and torsion angles; correlations between esds in cell parameters are only used when they are defined by crystal symmetry. An approximate (isotropic) treatment of cell esds is used for estimating esds involving l.s. planes.
Refinement. Refinement of F^2^ against ALL reflections. The weighted R-factor wR and goodness of fit S are based on F^2^, conventional R-factors R are based on F, with F set to zero for negative F^2^. The threshold expression of F^2^ > 2sigma(F^2^) is used only for calculating R-factors(gt) etc. and is not relevant to the choice of reflections for refinement. R-factors based on F^2^ are statistically about twice as large as those based on F, and R- factors based on ALL data will be even larger.

### Fractional atomic coordinates and isotropic or equivalent isotropic displacement parameters (Å^2^)

**Table:**

	*x*	*y*	*z*	*U*_iso_*/*U*_eq_	
C1	0.2321 (3)	0.5749 (3)	0.10380 (9)	0.0350 (4)	
C2	0.3128 (3)	0.3529 (3)	0.06412 (9)	0.0393 (4)	
H2	0.2234	0.3183	0.0257	0.047*	
C3	0.5197 (3)	0.1956 (3)	0.08329 (9)	0.0397 (4)	
H3	0.5754	0.0493	0.0580	0.048*	
C4	0.6579 (3)	0.2491 (3)	0.14265 (9)	0.0341 (4)	
C5	0.6847 (3)	0.5467 (3)	0.23871 (9)	0.0382 (4)	
H5A	0.8698	0.4820	0.2316	0.046*	
H5B	0.6670	0.7278	0.2356	0.046*	
C6	0.6498 (5)	0.2102 (4)	0.32915 (12)	0.0650 (6)	
H6A	0.8343	0.1520	0.3166	0.078*	
H6B	0.5510	0.1163	0.3018	0.078*	
C7	0.5809 (7)	0.1687 (6)	0.40974 (14)	0.0896 (9)	
H7A	0.6172	−0.0086	0.4221	0.108*	
H7B	0.6888	0.2541	0.4367	0.108*	
C8	0.2589 (6)	0.5165 (7)	0.41244 (14)	0.0896 (9)	
H8A	0.3627	0.6065	0.4394	0.107*	
H8B	0.0761	0.5767	0.4269	0.107*	
C9	0.3172 (4)	0.5693 (5)	0.33187 (11)	0.0609 (6)	
H9A	0.2066	0.4883	0.3046	0.073*	
H9B	0.2821	0.7480	0.3214	0.073*	
N1	0.3486 (3)	0.6309 (2)	0.15815 (7)	0.0350 (3)	
N2	0.5574 (2)	0.4640 (2)	0.17747 (7)	0.0326 (3)	
N3	0.5895 (3)	0.4751 (3)	0.31060 (8)	0.0430 (4)	
O1	0.0275 (2)	0.7323 (2)	0.08280 (7)	0.0511 (4)	
H1	−0.0028	0.8530	0.1092	0.077*	
O2	0.8565 (2)	0.1128 (2)	0.16258 (7)	0.0484 (4)	
O3	0.3137 (5)	0.2588 (5)	0.43038 (10)	0.1016 (7)	

### Atomic displacement parameters (Å^2^)

**Table:**

	*U*^11^	*U*^22^	*U*^33^	*U*^12^	*U*^13^	*U*^23^
C1	0.0322 (8)	0.0339 (8)	0.0352 (8)	0.0063 (6)	−0.0032 (6)	−0.0019 (6)
C2	0.0397 (9)	0.0387 (9)	0.0382 (9)	0.0042 (7)	−0.0095 (7)	−0.0088 (7)
C3	0.0432 (10)	0.0327 (9)	0.0403 (9)	0.0070 (7)	−0.0066 (7)	−0.0111 (7)
C4	0.0337 (8)	0.0291 (8)	0.0366 (8)	0.0037 (6)	−0.0019 (6)	−0.0027 (6)
C5	0.0377 (9)	0.0374 (9)	0.0411 (9)	−0.0050 (7)	−0.0100 (7)	−0.0064 (7)
C6	0.0911 (17)	0.0508 (13)	0.0530 (12)	−0.0103 (12)	−0.0110 (11)	0.0064 (10)
C7	0.132 (3)	0.0806 (19)	0.0567 (15)	−0.0218 (18)	−0.0130 (16)	0.0188 (13)
C8	0.0836 (19)	0.125 (3)	0.0571 (15)	−0.0191 (18)	0.0134 (13)	−0.0112 (16)
C9	0.0515 (12)	0.0807 (16)	0.0503 (12)	−0.0107 (11)	0.0010 (9)	−0.0100 (11)
N1	0.0349 (7)	0.0299 (7)	0.0373 (7)	0.0065 (5)	−0.0063 (6)	−0.0043 (5)
N2	0.0314 (7)	0.0292 (7)	0.0353 (7)	0.0039 (5)	−0.0066 (5)	−0.0042 (5)
N3	0.0480 (9)	0.0448 (9)	0.0375 (8)	−0.0066 (7)	−0.0093 (6)	−0.0049 (6)
O1	0.0495 (8)	0.0477 (8)	0.0504 (8)	0.0227 (6)	−0.0192 (6)	−0.0131 (6)
O2	0.0417 (7)	0.0458 (7)	0.0523 (8)	0.0175 (6)	−0.0137 (6)	−0.0091 (6)
O3	0.1169 (18)	0.1262 (19)	0.0662 (12)	−0.0507 (15)	0.0097 (11)	0.0167 (12)

### Geometric parameters (Å, º)

**Table:**

C1—N1	1.297 (2)	C6—H6A	0.9700
C1—O1	1.3341 (18)	C6—H6B	0.9700
C1—C2	1.421 (2)	C7—O3	1.419 (4)
C2—C3	1.331 (2)	C7—H7A	0.9700
C2—H2	0.9300	C7—H7B	0.9700
C3—C4	1.437 (2)	C8—O3	1.410 (4)
C3—H3	0.9300	C8—C9	1.509 (3)
C4—O2	1.2497 (18)	C8—H8A	0.9700
C4—N2	1.360 (2)	C8—H8B	0.9700
C5—N3	1.425 (2)	C9—N3	1.449 (3)
C5—N2	1.4892 (19)	C9—H9A	0.9700
C5—H5A	0.9700	C9—H9B	0.9700
C5—H5B	0.9700	N1—N2	1.3660 (17)
C6—N3	1.452 (3)	O1—H1	0.8200
C6—C7	1.508 (3)		
			
N1—C1—O1	119.33 (14)	C6—C7—H7A	109.4
N1—C1—C2	123.26 (14)	O3—C7—H7B	109.4
O1—C1—C2	117.41 (14)	C6—C7—H7B	109.4
C3—C2—C1	118.37 (15)	H7A—C7—H7B	108.0
C3—C2—H2	120.8	O3—C8—C9	111.8 (2)
C1—C2—H2	120.8	O3—C8—H8A	109.3
C2—C3—C4	120.80 (14)	C9—C8—H8A	109.3
C2—C3—H3	119.6	O3—C8—H8B	109.3
C4—C3—H3	119.6	C9—C8—H8B	109.3
O2—C4—N2	120.60 (14)	H8A—C8—H8B	107.9
O2—C4—C3	124.21 (14)	N3—C9—C8	108.85 (19)
N2—C4—C3	115.19 (13)	N3—C9—H9A	109.9
N3—C5—N2	116.93 (13)	C8—C9—H9A	109.9
N3—C5—H5A	108.1	N3—C9—H9B	109.9
N2—C5—H5A	108.1	C8—C9—H9B	109.9
N3—C5—H5B	108.1	H9A—C9—H9B	108.3
N2—C5—H5B	108.1	C1—N1—N2	117.12 (12)
H5A—C5—H5B	107.3	C4—N2—N1	125.20 (13)
N3—C6—C7	109.2 (2)	C4—N2—C5	121.09 (13)
N3—C6—H6A	109.8	N1—N2—C5	113.57 (12)
C7—C6—H6A	109.8	C5—N3—C9	115.28 (15)
N3—C6—H6B	109.8	C5—N3—C6	115.05 (15)
C7—C6—H6B	109.8	C9—N3—C6	110.95 (18)
H6A—C6—H6B	108.3	C1—O1—H1	109.5
O3—C7—C6	111.4 (2)	C8—O3—C7	109.8 (2)
O3—C7—H7A	109.4		
			
N1—C1—C2—C3	−0.6 (3)	C1—N1—N2—C4	2.5 (2)
O1—C1—C2—C3	178.72 (16)	C1—N1—N2—C5	178.12 (14)
C1—C2—C3—C4	0.0 (3)	N3—C5—N2—C4	−94.05 (18)
C2—C3—C4—O2	−178.44 (17)	N3—C5—N2—N1	90.12 (17)
C2—C3—C4—N2	1.7 (2)	N2—C5—N3—C9	−61.2 (2)
N3—C6—C7—O3	57.7 (3)	N2—C5—N3—C6	69.9 (2)
O3—C8—C9—N3	−58.1 (3)	C8—C9—N3—C5	−170.26 (19)
O1—C1—N1—N2	−179.85 (14)	C8—C9—N3—C6	56.7 (2)
C2—C1—N1—N2	−0.5 (2)	C7—C6—N3—C5	170.00 (19)
O2—C4—N2—N1	177.10 (14)	C7—C6—N3—C9	−56.9 (3)
C3—C4—N2—N1	−3.0 (2)	C9—C8—O3—C7	59.2 (3)
O2—C4—N2—C5	1.8 (2)	C6—C7—O3—C8	−58.9 (3)
C3—C4—N2—C5	−178.32 (14)		

### Hydrogen-bond geometry (Å, º)

**Table:**

*D*—H···*A*	*D*—H	H···*A*	*D*···*A*	*D*—H···*A*
O1—H1···O2^i^	0.82	1.77	2.5777 (16)	167
C2—H2···O1^ii^	0.93	2.55	3.478 (2)	175
C5—H5*A*···O2	0.97	2.44	2.772 (2)	100

Symmetry codes: (i) *x*−1, *y*+1, *z*; (ii) −*x*, −*y*+1, −*z*.
